# Supplementary material for: Identification of photoperiod-induced specific miRNAs in the adrenal glands of Sunite sheep (Ovis aries)
Source: Front Vet Sci. 2022 Jul 22;9:888207. doi: 10.3389/fvets.2022.888207 (PMC9354845; doi:10.3389/fvets.2022.888207)
Supplement: Supplementary file 1 [file Data_Sheet_1.ZIP › Supplementary file/miRDeep2 file.zip!/miRDeep2 file/Novel_Structure/SPLP42A2/Novel_502.pdf]

Provisional ID	:	NC_019484.2_43624
Score total	:	1.6
Score for star read(s)	:	-1.3
Score for read counts	:	0
Score for mfe	:	1.3
Score for randfold	:	1.6
Score for cons. seed	:	
Total read count	:	137
Mature read count	:	137
Loop read count	:	0
Star read count	:	0

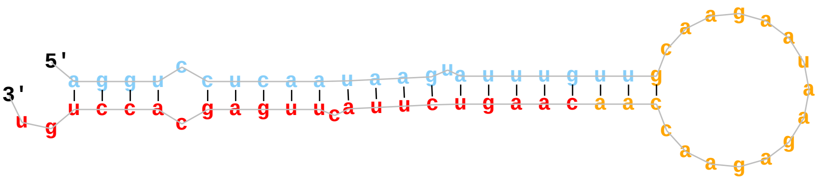

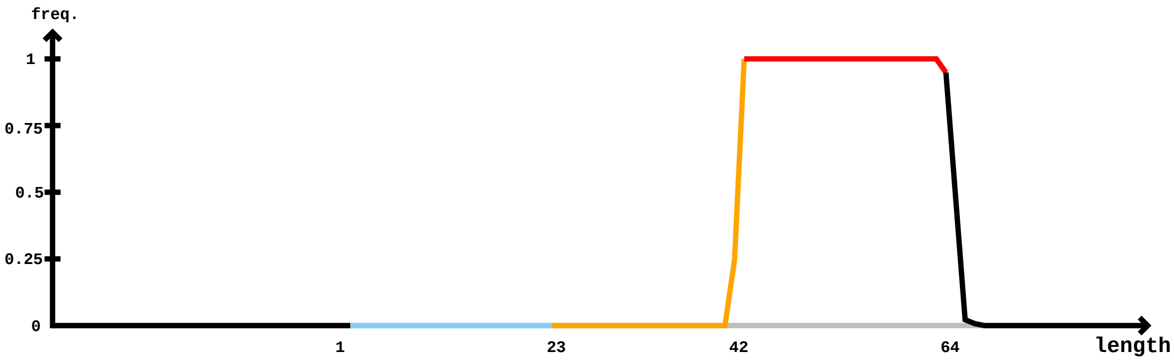


```
                                   Star                                      Mature
5'- auuguguuucuucaugccugacauauagcagguccucaauaaguauuuguugcaagaauaagagaaccaacaagucuuacuugagcaccuguuauguggaggauaaaaagcu   -3'    exp
    .....((((.((.((.(((..((((((((((((.(((((((((.((((((((..............)))))))))))).))))).))))))))))))).))))))).)))).   reads      mm         sample
    .....................................................................acaagucuuacuugagcaccug.....................   2          0          seq
    .....................................................................acaagucuuacuugagcaccugu....................   27         0          seq
    .....................................................................acaagucuuacuugagcaccuguu...................   4          0          seq
    .....................................................................acaagucuuacuugagcaccuguuau.................   1          0          seq
    ......................................................................caagucuuacuugagcaccug.....................   5          0          seq
    ......................................................................caagucuuacuugagcaccugu....................   83         0          seq
    ......................................................................caagucuuacuugagcaccuguu...................   13         0          seq
    ......................................................................caagucuuacuugagcaccuguua..................   2          0          seq
```
